# Targeting endosialin/CD248 through antibody-mediated internalization results in impaired pericyte maturation and dysfunctional tumor microvasculature

**DOI:** 10.18632/oncotarget.4559

**Published:** 2015-07-04

**Authors:** Katherine Rybinski, Hongxia Z. Imtiyaz, Barrie Mittica, Brian Drozdowski, James Fulmer, Keiji Furuuchi, Shawn Fernando, Marianne Henry, Qimin Chao, Brad Kline, Earl Albone, Jason Wustner, JianMin Lin, Nicholas C. Nicolaides, Luigi Grasso, Yuhong Zhou

**Affiliations:** ^1^ Morphotek, Inc., Exton, PA 19341, USA

**Keywords:** endosialin, CD248, MORAb-004, tumor microvasculature, α-SMA

## Abstract

Over-expression of endosialin/CD248 (herein referred to as CD248) has been associated with increased tumor microvasculature in various tissue origins which makes it an attractive anti-angiogenic target. In an effort to target CD248, we have generated a human CD248 knock-in mouse line and MORAb-004, the humanized version of the mouse anti-human CD248 antibody Fb5. Here, we report that MORAb-004 treatment significantly impacted syngeneic tumor growth and tumor metastasis in the human CD248 knock-in mice. In comparison with untreated tumors, MORAb-004 treated tumors displayed overall shortened and distorted blood vessels. Immunofluorescent staining of tumor sections revealed drastically more small and dysfunctional vessels in the treated tumors. The CD248 levels on cell surfaces of neovasculature pericytes were significantly reduced due to its internalization. This reduction of CD248 was also accompanied by reduced α-SMA expression, depolarization of pericytes and endothelium, and ultimately dysfunctional microvessels. These results suggest that MORAb-004 reduced CD248 on pericytes, impaired tumor microvasculature maturation and ultimately suppressed tumor development.

## INTRODUCTION

Angiogenesis plays an essential role not only in embryonic development and wound healing, but also in cancer development [1, 2]. In the past thirty years, studies of angiogenesis, the different participating cell types and proteins have yielded multiple attractive therapeutic targets [3]. The first of such targets, VEGFR, is expressed on vascular endothelial cells [4]. Intensive studies of this gene have led to the successful launch of an anti-VEGFR antibody and a VEGF-trap, the first two anti-angiogenesis therapeutic agents for metastatic cancers and age-related macular degeneration (AMD) [5]. Another target, PDGFRβ, is expressed on pericytes surrounding the endothelial cells and has been targeted with both antibodies and small molecule agents [6]. An anti-PDFGR antibody (Fovista^®^) has been advanced to the clinical stage and recently had a successful Phase 2b clinical trial in combination with an anti-VEGF agent for the treatment of patients newly diagnosed with wet AMD [7]. These successes have stimulated the search for more angiogenesis targets expressed directly on endothelial cells as well as stromal cells that support vessel formation. One such protein, CD248, is expressed on tumor associated pericytes and tumor stromal fibroblasts and was identified as a potential anti-tumor vasculature target due to its tight association with tumor neovasculature [3].

CD248 was originally discovered as a human embryonic fibroblast specific antigen reactive to antibody Fb5 and was thought to be selectively expressed on the vascular endothelial cells of malignant tumors, thus named endosialin [8]. The same gene was rediscovered as TEM-1 (for tumor endothelial marker-1) by global gene analysis of tumor vessels isolated from primary colorectal tumor tissue using SAGE analysis [9]. Subsequent and more refined studies found CD248 was specifically expressed on tumor-associated pericytes and stromal cells, but not directly by tumor-associated endothelial cells [10, 11]. Independent studies have found CD248 mRNA or protein to be highly associated with multiple human cancers including colorectal, breast, histiocytomas, highly invasive glioblastoma, anaplastic astrocytomas, and metastatic melanomas [3].

Although the expression of CD248 is well documented, its biological function is less clear. Our laboratory and others have reported that CD248 interacts with multiple extracellular matrix proteins and mediates pericyte proliferation [12, 13]. So far only one publication demonstrated that CD248 is directly involved in tumor neovascularization [14]. In this report, the authors showed that in comparison with tumors in parental animals, tumor growth, invasion, and metastasis were drastically reduced in CD248 knockout mice. Tumors that grew in the knockout mice also showed abnormal blood vessel sizes and structure, suggesting that CD248 functionally governed the proper growth and formation of tumor vessels [14]. This result prompted us to test anti-CD248 antibody activity on tumor growth and metastasis in a therapeutic setting. The anti-CD248 antibodies generated to date, including MORAb-004, have been human specific therefore limiting the ability to test the biological activity of blocking CD248 in standard human cell-derived mouse xenograft models. In order to test MORAb-004 biological activity on tumor growth we generated a human CD248 knock-in mouse line and evaluated the effect of MORAb-004 in syngeneic tumor models.

## RESULTS

### MORAb-004 treatment reduced CD248 expression on pericytes by inducing its internalization

**Figure 1:**
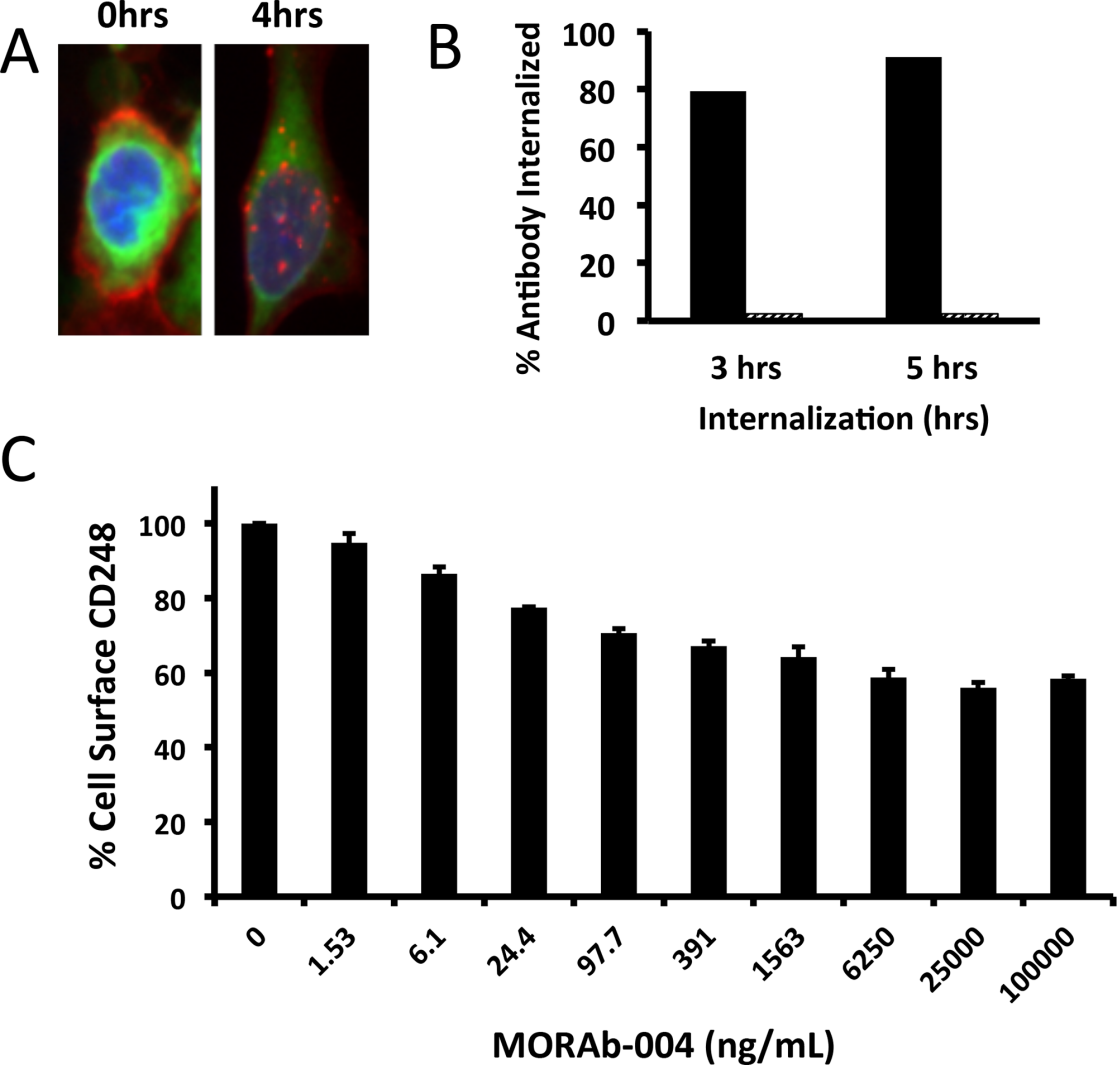
Internalization of MORAb-004 and CD248 on human pericytes **A.** IF images of human pericytes treated with MORAb-004; plasma membrane dye in green, AlexaFluor-555 conjugated anti-CD248 in red. **B, C.** Internalization assays of *in vitro* cultured human pericytes: (B) shows the percentage of internalized MORAb-004 (solid bars) or monoclonal IgG isotype control (hashed bars); (C) shows the percentage of reduction of surface CD248 upon MORAb-004 treatment for 12 hrs.

MORAb-004 is a high affinity, humanized antibody to CD248 that has shown a good CD248 binding via flow cytometry [14]. While highly specific for CD248, MORAb-004 lacks immune-effector related functions such as antibody dependent cell-mediated cytotoxicity (ADCC) or complement dependent cytotoxicity (CDC) on CD248-expressing cells (Figure S1A, S1B). Rather, immunofluorescent staining of MORAb-004 treated primary human pericytes revealed that this antibody is quickly internalized (Figure 1A). Antibody internalization reached its maximum after 3–5 hours incubation and 80% of MORAb-004 was detected in the cytoplasm (Figure 1B). To examine whether MORAb-004 treatment would directly cause reduction of CD248 levels on the cell surface, pericytes were cultured in the presence of MORAb-004 for up to 18 hours and then stained with a labeled anti-CD248 monoclonal IgG, 9G5, which recognizes a different epitope from that of MORAb-004 (Figure S1C). This assay permitted measurement of residual surface CD248 expression in pericytes after treatment with MORAb-004. The results of this assay demonstrated a MORAb-004 dose-dependent reduction of up to 46% of cell surface CD248 (Figure 1C).

### Validation of human CD248 knock-in mice

A human CD248 knock-in mouse line was created on the C57BL/6 background to overcome the challenge of lack of cross-reactivity of MORAb-004 with murine CD248 (Figure S2A). Genotyping of the homozygous knock-in mice confirmed the correct replacement of mouse CD248 locus with human CD248 gene and RT-PCR confirmed that no mouse CD248 gene expression was detectable (Figure S2B).

The homozygous human CD248 (huCD248) knock-in mice were healthy with no gross differences compared to their wild type littermates. To verify the proper expression of the human gene in the knock-in mice, tissues from eight major organs of huCD248 knock-in mice and wild type C57BL/6 mice were collected and subjected to Western blot analysis. While mCD248 expression levels vary among all exanimated tissues, huCD248 expression levels in the huCD248 knock-in mice mirrored those in the paired wild type mice (Figure S2C). Immunofluorescent staining of normal lung sections of C57BL/6 mice showed that murine CD248 was highly expressed on fibroblastoid-like cells loosely surrounding the large blood vessels (Figure S3A left, red arrow) but absent in vascular smooth muscle cells tightly surrounding the endothelium of those blood vessels (Figure S3A, white arrow). Similar expression patterns were seen for human CD248 in knock-in mice (Figure S3A, right lower panel). It has been reported that expression of human CD248 is highly induced on the pericytes surrounding the newly formed tumor blood vessels [3]. When immunofluorescent staining was performed on B16-F10 sc tumor sections, human CD248 was found highly expressed on the outer layer cells of the neovasculature (Figure S3B). The same cells also expressed alpha smooth muscle actin (α-SMA) and are tightly associated with the CD31-expressing endothelial cells, suggesting their pericyte origin (Figure S3B). Collectively, this study suggests that the huCD248 knock-in gene fully retained the expression pattern of the endogenous mouse gene.

### MORAb-004 significantly impacted primary tumor growth and tumor metastasis

**Figure 2:**
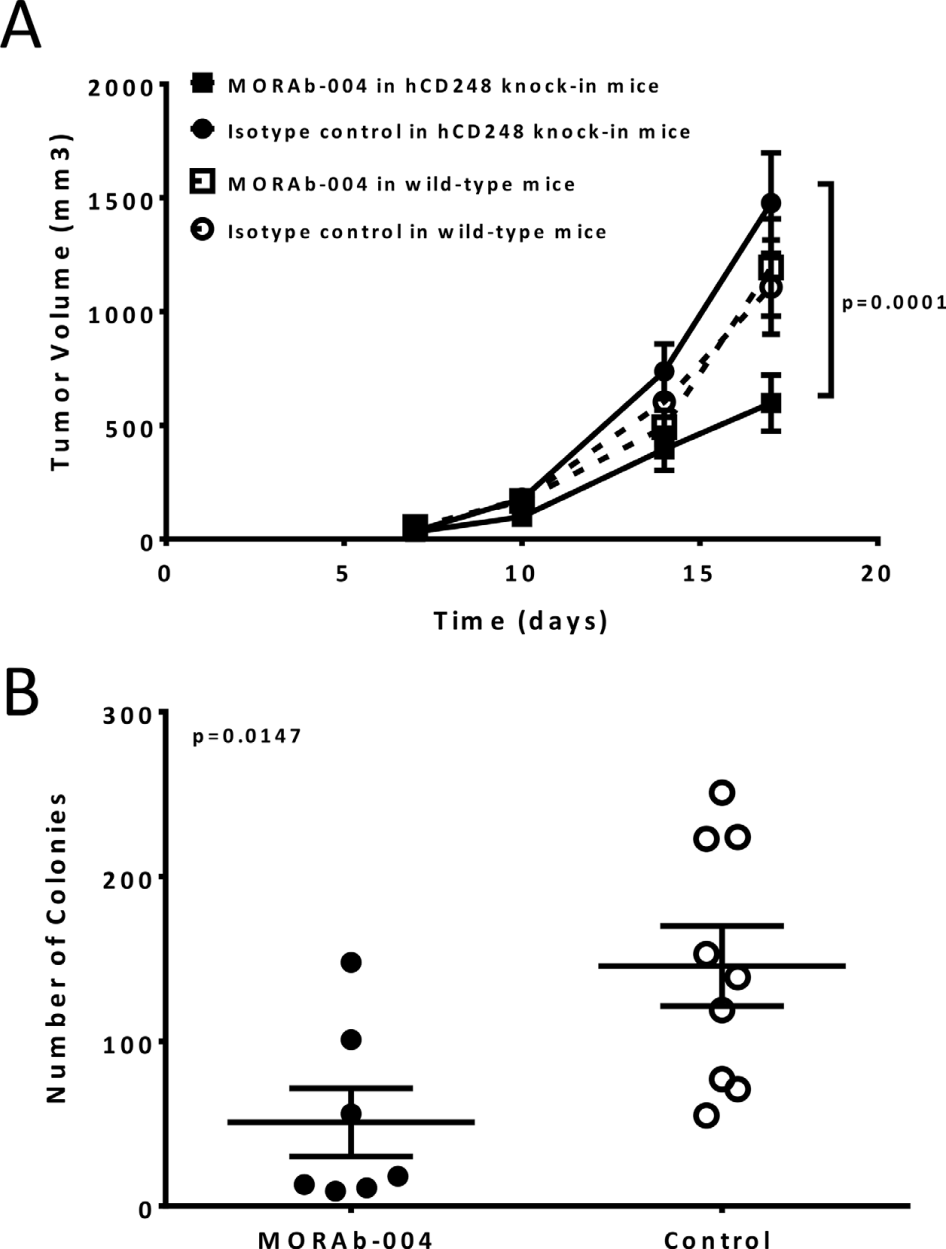
MORAb-004 inhibited B16-F10 tumor progression and lung colonization only in mice expressing human CD248 **A.** B16-F10 cells, at 5 × 10^5^, were injected s.c. into right flank of the huCD248 knock-in mice or syngeneic C57BL/6 wild type mice (*n* = 16). MORAb-004 or a monoclonal IgG isotype control antibody, were administered i.v. via tail vein at 50 mg/kg, 5 doses daily, starting on day 3 post tumor cell implantation. Tumor growth was monitored twice weekly starting on day 7 by three dimensional caliper measurement and presented by volume (mm^3^). Data presented as Mean ± SEM. **B.** B16-F10-L1 cells, at 1 × 10^5^, were injected i.v. via tail vein into the huCD248 knock-in mice (*n* = 16). MORAb-004 or a monoclonal IgG isotype control antibody was administered i.v. via tail vein at 50 mg/kg, 1 day prior to tumor cell injection and every other day post implantation for a total of 5 doses. At the end of the study (day 19 post tumor cell implantations) mouse lungs were harvested. Black-colored melanoma colonies were counted and colony numbers of every mouse were graphed as tumor burden. *P* = 0.012 (*t*-test).

Studies in murine CD248 knockout mice had shown that CD248 played an essential role on tumor growth [14]. To confirm whether CD248 targeting with an antibody will have similar effect as CD248 knockout, MORAb-004 activity was evaluated using both a B16-F10 subcutaneous model and a lung colonization model. In the subcutaneous model, B16-F10 cells were injected into either huCD248 knock-in mice or C57BL/6 wild type mice. Administration of 50 mg/kg MORAb-004 for 5 consecutive days starting at 3 days after tumor implantation reduced tumor growth approximately 70% (by volume) compared to that in the control animals (*P* value < 0.01, Figure 2A). In the same study, MORAb-004 showed no effect on tumor growth when given to C57BL/6 wild type mice bearing the same tumors (Figure 2A).

To determine the effect of MORAb-004 treatment on tumor cell dissemination and colonization that induce distant lesions, B16-F10 melanoma cells adapted for lung colonization (B16-F10-L1) were injected intravenously into the huCD248 knock-in mice. One day prior to cell implantation, mice were treated with 50mg/kg MORAb-004 or a control antibody followed by a regimen of additional doses every other day after tumor cell implantation for 4 doses. On day 19, melanoma colonies in the lungs were counted and recorded as tumor burden. MORAb-004 treatment reduced lung colonization by approximately 70% compared to the control animals (Figure 2B, *P* value < 0.01). A similar model, where Lewis lung carcinomas colonize in mouse lungs, was explored to confirm the effect of MORAb-004 treatment. Similar to the observations in the B16-F10 model, MORAb-004 treatment significantly reduced tumor cell lung colonization as compared to that in the control treatment (Figure S4A) (*P* value < 0.01). Fb5 (a fully mouse MORAb-004 precursor antibody) was used in the same study and demonstrated a more pronounced effect in inhibiting tumor cell colonization (Figure S4B). These results indicate that MORAb-004 could not only reduce primary tumor growth, but also inhibit tumor metastasis.

### MORAb-004 treated tumors contained significantly greater numbers of microvessels that were small and nonfunctional

**Figure 3:**
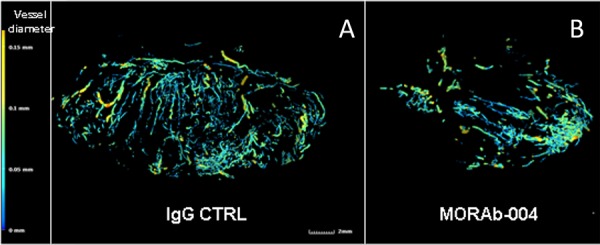
Microfill perfusion and x-ray micro-CT angiography The tumor bearing mice in either the MORAb-004 treated or control group were perfused with sodium nitroprusside containing solution followed by MICROFIL (Carver) injections. The tumors retaining polymerized latex were fixed in formalin then imaged with a X-ray micro-CT system performed by Numira Biosciences, and analyzed using an image analysis software package (Altaview, Numira). Microfill perfusion of animals show extensive neo-vascularization within the tumor of CTRL treated animals **A.** while MORAb-004 treatment **B.** reduced the amount of new vessels within the developing tumor.

**Figure 4:**
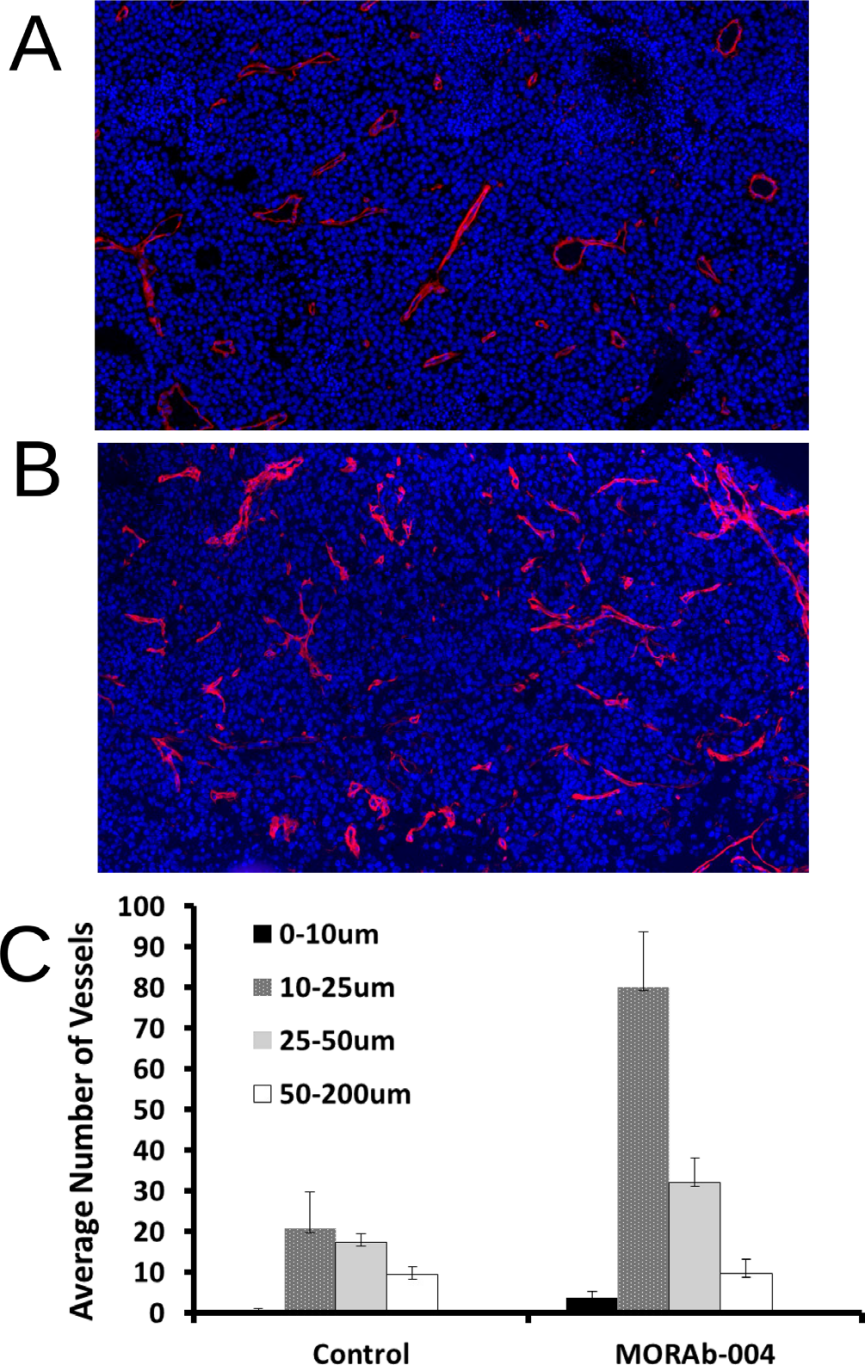
Immunofluorescent staining and digital analysis of tumor microvessels **A.** PBS treated (control) B16-F10 s.c. tumor section stained with Collagen IV (ColIV); **B.** MORAb-004 treated B16-F10 s.c. tumor section stained with ColIV; **C.** Comparison of digital counting of microvessels grouped by size. All images were captured at 20x using Panoramic Midi digital slide scanner.

To examine whether there is any effect of CD248 disruption via MORAb-004 antibody treatment on tumor microvasculature, microfill perfusion and X-ray micro-CT angiography was performed on animals of each treatment group. The result of this technology provided 3D images of the entire functional vasculature network inside a tumor. A dense vasculature network with extensive neo-vascularization (vessel size < 50 um) was seen within the tumors of control mice (Figure 3A). In sharp contrast, MORAb-004 treated tumors exhibited a drastic reduction in the amount of small, new vessels (<50 um) with most of the functional vessels (50–100 um) exhibiting abrupt truncation (Figure 3B). To examine the microscopic structure of the tumor microvasculature in more detail, immunofluorescent staining was performed on representative tumor sections where Collagen IV staining outlined tumor blood vessels [15]. Overall, most microvessels in control tumors appeared to be functional with a defined open lumen that contained erythrocytes. Surprisingly, MORAb-004 treated tumors contained significantly greater numbers of microvessels, but most of them are devoid of erythrocytes (Figure 4A, 4B).

Because of the inherent heterogeneity of tumors and tumor microvessels, stained sections were digitally scanned and analyzed to achieve a better objective assessment of the differences between treatment groups. At a minimum, two representative tumors from each treatment group of each model were used and three images of comparative area (size of 20 × 30 μm^2^) were analyzed from each tumor for quantification of blood vessel numbers and sizes. Digital quantification revealed that compared to control tumors, not only were there more blood vessels present (Figure S5), but also significantly more small vessels (<50 um diameter) in treated tumors (Figure 4C). Similar differences were observed when blood vessels were stained with CD31 to highlight the endothelial content of the microvessels (Data not shown).

### MORAb-004 treatment resulted in reduction of CD248 and α-SMA levels on neovasculature pericytes

**Figure 5:**
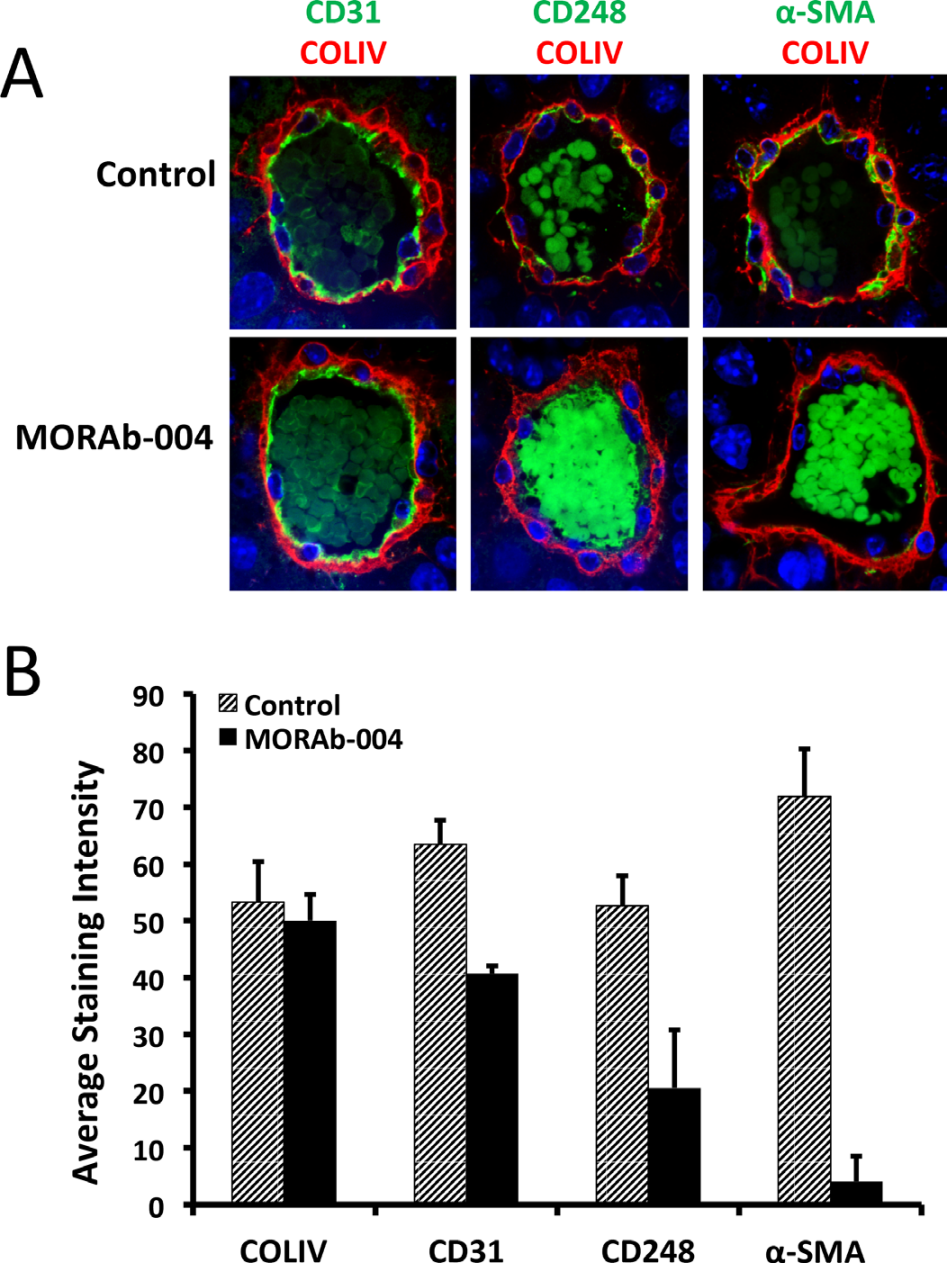
Immunofluorescent staining and digital analysis of CD248 expression levels and other markers on tumor microvessels **A.** IF images of B16-F10 tumor microvessels stained with CD31, CD248, and α-SMA (green), co-stained with ColIV (red). All images were captured at 60X using Olympus IX81confocal microscope; **B.** Comparison of relative expression levels of IF staining of markers on monoclonal isotype control and MORAb-004 treated microvessels.

Analysis of tumors from xenografts grown in CD248 knockout mice found that these tumors contained a significantly increased number of smaller vessels (<50 μm diameter) in comparison to those tumors from wild type mice, suggesting CD248 expression on tumor microvessels is essential for vessel growth and maturation [14]. The strikingly similar microscopic footprint of MORAb-004 treated tumors as compared to tumors from CD248 knockout mice, suggests that MORAb-004 might exert its impact through down-regulation of CD248 levels on microvessel pericytes. Although MORAb-004 can cause internalization and reduction of CD248 levels on *in vitro* cultured human brain pericytes, its effect on cell surface CD248 levels of tumor microvessels has not been previously documented. To examine the impact of MORAb-004 treatment on cell surface CD248 levels of tumor microvessels, two-color immunofluorescent staining on tumor sections was performed with Collagen IV (ColIV) in red and either CD248 or α-SMA (pericyte markers) or CD31 (endothelium marker) in green. In control tumors, ColIV and CD31staining clearly outlined the double layers of cells that constructed microvessels, where CD31 stained the inner layer endothelial cells and ColIV stained the outer layer cells (Figure 5A, upper left panel). Co-staining of ColIV with CD248 or α-SMA showed complete co-localization of these three proteins on the outer layer cells indicating that those cells were indeed pericytes (Figure 5A, upper middle and right panel). When the same panel of co-staining was performed on MORAb-004 treated tumors, the staining images of the few functional microvessels (the ones with an open lumen that contained erythrocytes) showed that, although the similarly ColIV-coated pericyte layer was intact on these microvessels outlining CD31-expressing endothelium (Figure 5A, lower left panel), CD248 and α-SMA expression on those cells were either negative or significantly reduced (Figure 5A, lower middle and right panel).

Digital quantification of ColIV staining revealed that there were no apparent differences in average intensity of ColIV staining on tumor microvessels between control and treated groups. Similar quantification of CD31 staining revealed that there was modest reduction in CD31 levels in the MORAb-004 treated vessels compared to those of the controls. Quantification of CD248 staining revealed that there was a drastic reduction of CD248 levels on MORAb-004 treated microvessels compared to the level on control vessels. The reduced CD248 levels were accompanied by an even greater reduction in α-SMA levels on MORAb-004 treated microvessels (Figure 5B).

### MORAb-004 treatment caused internalization of CD248 and depolarization of pericytes and endothelial cells

**Figure 6:**
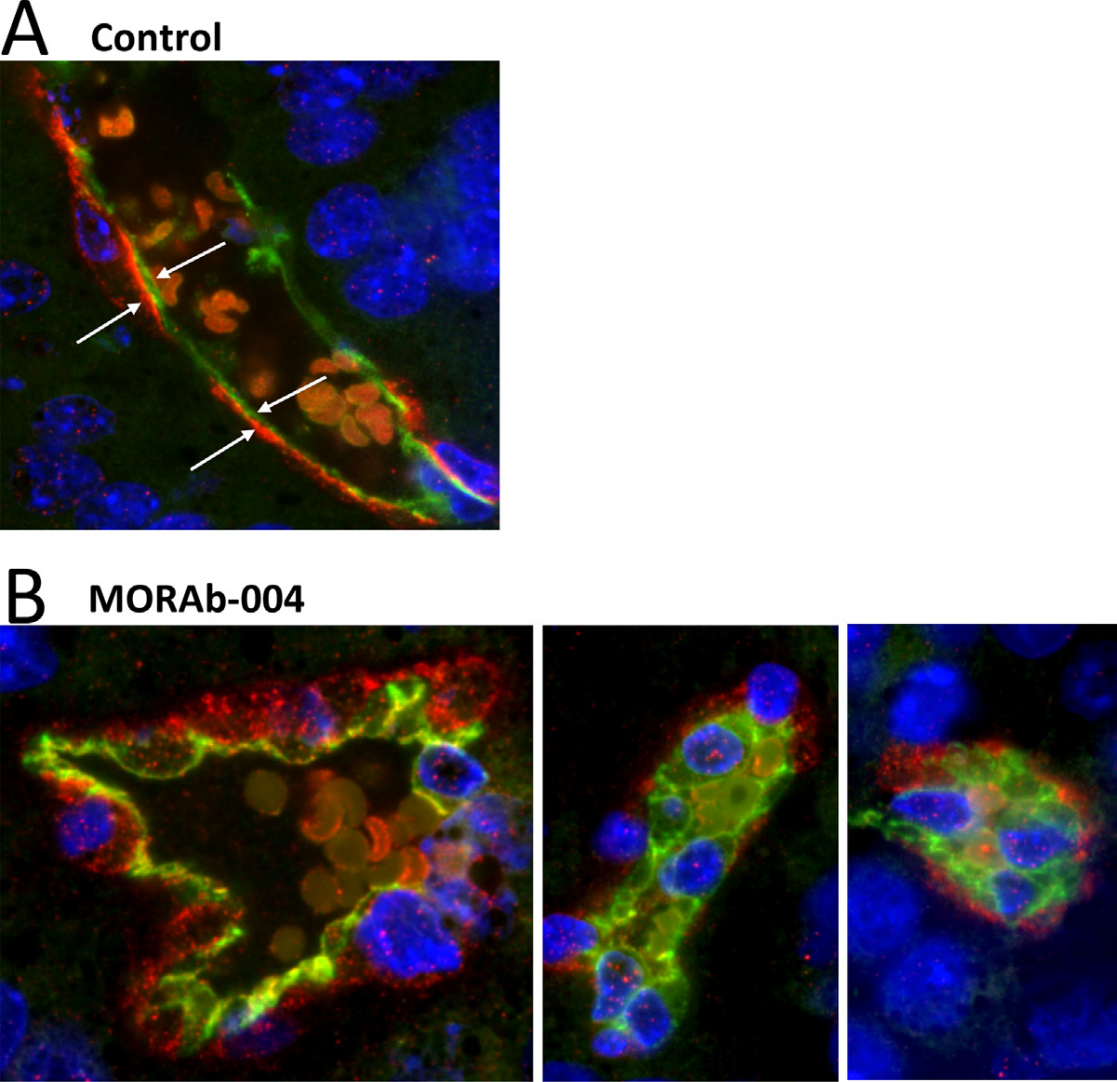
MORAb-004 treatment caused internalization of CD248 and depolarization of pericytes and endothelium IF images of control (PBS) or MORAb-004 treated B16-F10 tumor micro-vessels stained for CD31 (green) and CD248 (red). All images were captured at 60X using an Olympus IX81confocal microscope.

Not only were both CD248 and α-SMA co-expressed on pericytes on control tumor microvessels, their cellular distribution patterns were similarly polarized to the basal side of pericytes towards the endothelium (Figure S3B, 5A and 6A). Interestingly, CD31 expression on endothelial cells was also polarized toward the basal side of the pericytes (Figure S3B, 5A and 6A) where CD31 seemed to be proximal and form physical associations with CD248 and α-SMA. However, in the MORAb-004 treated microvessels, even in those few pericytes with residual CD248 expression, massive internalization of CD248 protein and depolarization of its cellular distribution were very distinct from the controls (Figure 6B). On those more severely affected vessels with closed lumen, CD31 also lost its polarized distribution, and both endothelial cells and pericytes displayed a rounded and depolarized morphology (Figure 6B, middle and right panels).

### CD248 expression is required for TGF-β induced α-SMA expression on pericytes

**Figure 7:**
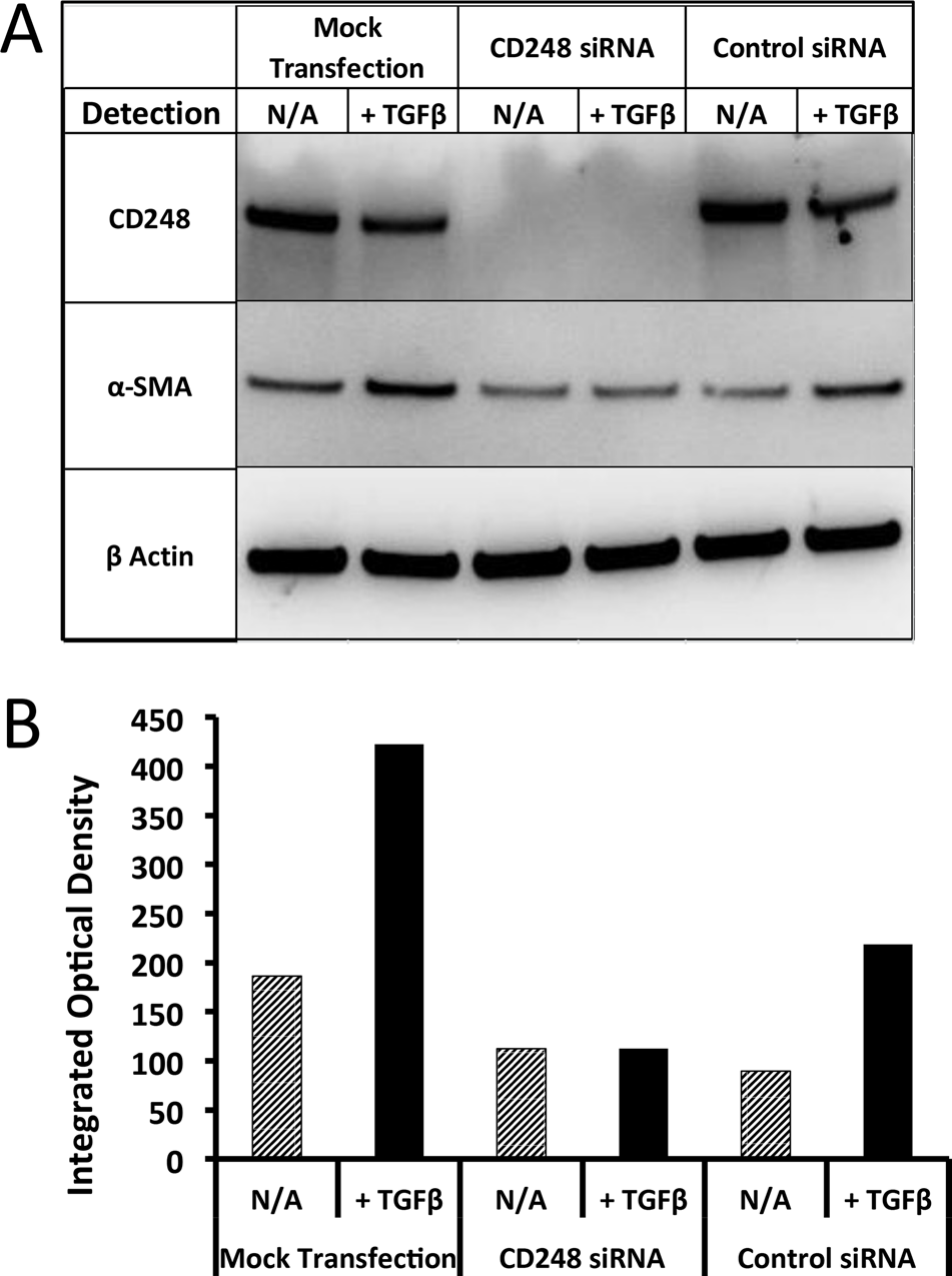
Induction of α-SMA in human pericytes and suppression effects of targeting CD248 **A.** Western blotting analysis of CD248, α-SMA expression on CD248 siRNA transfected human pericytes; **B.** Western blotting image quantification.

The reduced α-SMA expression on MORAb-004 treated tumor blood vessels could be either a mere association with reduced CD248 expression or a direct consequence of CD248 reduction, meaning CD248 directly regulates α-SMA expression on pericytes. To test the latter hypothesis, human pericytes were transfected with either CD248 specific siRNA (#2163) or specific control siRNA (#2163-C911) and both CD248 and α-SMA levels in transfected cells were measured. As shown in Figure 7A, CD248 specific siRNA (#2163) reduced CD248 expression in these cells to undetectable levels, while specific control siRNA (#2163–C911) had no impact on CD248 expression levels. As for α-SMA, there were basal levels of expression in all test conditions, and α-SMA expression could be further induced when the mock transfected or control siRNA transfected pericytes were treated with TGF-β. However this induction of α-SMA expression was completely absent in the CD248 specific siRNA transfected cells (Figure 7A, 7B), suggesting that α-SMA induction is CD248 dependent.

Taken together, our data demonstrated that MORAb-004 treatment induced internalization and degradation of CD248 on pericytes, which in turn altered the α-SMA expression pattern. All these molecular changes eventually caused morphological changes and potential malfunctions of both pericytes and endothelial cells during angiogenesis.

## DISCUSSION

CD248 is structurally classified as a C-type lectin-like protein, composed of five globular extracellular domains and a short cytoplasmic tail with no apparent phosphorylation sites [3]. The discovery of the functional importance of CD248 in tumor neovascularization has stimulated intense efforts in searching for CD248 interacting proteins. However, despite identification of a rich list of extracellular binding partners, the interaction that is critically responsible for angiogenesis remains to be elucidated [12, 16]. Since it is unclear which ligand interaction or multiple interactions collectively may be required in the process of angiogenesis, the multi-domain architecture and multiple interacting partners of CD248 provide a significant challenge to generate a single therapeutic agent that can effectively block the biological functions of CD248 and exert similar impact on tumor development as reported in the CD248 null mouse. In this study, we have discovered that MORAb-004, via its target antigen internalization function, was able to create an effect similar to that of CD248-null mouse in regards to tumor neovasculature. This property of MORAb-004 bypasses the necessity of an effective neutralizing antibody to destruct a specific ligand interaction; instead it exerted a much more profound effect by disrupting multiple critical ligand interactions. The finding that MORAb-004 lacks immune-effector activity (i.e. ADCC and CDC) suggests its effect is due to the disruption of the biological activity(s) of CD248 itself.

Analysis of MORAb-004-induced internalization and reduction of CD248 showed some inconsistences between human pericytes cultured *in vitro* and mouse pericytes derived from *in vivo* treated tumors. Despite quick internalization of 80% of surface bound MORAb-004 on *in vitro* cultured human pericytes, CD248 levels were reduced no more than 50% on those cells. In contrast, we observed an average 80% reduction of CD248 expression levels on MORAb-004 treated tumor pericytes, with the vast majority being completely negative for cell surface expression. The human pericytes used in the *in vitro* experiments were cultured in a specially optimized medium supplement with multiple growth factors (e.g. EGF and TGF-β). Under this optimal culture condition, pericytes stripped of surface CD248 could completely replenish CD248 within 5–6 hours (data not shown). This culture condition could also extend the life span of human pericytes to up to seven weeks or >10 passages [13]. In contrast, the B16-F10 tumors grew very aggressively where tumor size reached end point (>2000mm^3^) within two weeks after tumor cell inoculation. Concurrently, expression of CD248 dropped from the highest level on pericytes surrounding tumor microvessels to completely negative when the pericytes differentiated into smooth muscle cells as the blood vessels grew larger into a more artery-like structure. Since the *in vivo* pericytes might not need to retain high levels of CD248 for an extended period of time, the tumor microenvironment might lack the supporting factors for fast replenishment of CD248, which would explain why MORAb-004 could cause a more severe reduction *in vivo*.

The CD248 reduction caused by MORAb-004 treatment was associated with an even more significant α-SMA reduction. Our data from *in vitro* cultured human pericytes demonstrates that there were two mechanisms governing the expression of α-SMA in pericytes: a constant CD248-independent basal level expression and a TGF-β induced CD248-dependent expression. TGF-β is a pericyte growth factor secreted by endothelial cells. It not only mediates communication between the endothelial cells and pericytes, but the TGF-β signaling pathway also plays an important role in pericyte differentiation [17]. TGF-β induced α-SMA expression has been documented in kidney pericytes as well as lung fibroblastoid cells where CD248 expression is also present [18, 19]. It has been shown that TGF-β induces α-SMA expression through two pathways: the major pathway is through the ERK/MAPK and the minor pathway is through p38/MAPK [18]. We have previously reported that the ERK/MAPK pathway is involved in PDGF induced CD248 mediated pericyte proliferation [13]. Because of the cell membrane location of both CD248 and growth factor receptors, it is plausible to investigate whether there exists some direct interaction or cross-modifications of the two proteins. It remains to be discovered as to how exactly CD248 communicates with growth factor receptors and mediates cell signaling.

Taken together, our results revealed a mode of action of MORAb-004 by which internalization and reduction of CD248 on pericytes impaired the initiation of tumor microvessels and ultimately suppressed tumor angiogenesis.

## MATERIALS AND METHODS

### Cells and reagents

Primary human brain vascular pericytes were obtained from ScienCell (Carlsbad, CA) and grown according to the vendor's instructions. Human recombinant TGF-β was purchased from Peprotech (100-21). Antibodies used in the immunofluorescent staining were purchased from commercial sources as follows: rabbit-anti-mouse collagen IV (Millipore, AB756P), mouse-anti-mouse-α-SMA-FITC (Sigma, F3777), goat-anti-mouse CD31 (R&D, AF3628), Alexa Fluor 555 conjugated goat-anti-Rabbit Ig (Invitrogen, A21429) and Alexa Fluor 488 conjugated goat-anti-human Ig (Invitrogen, A11013). Two monoclonal anti-CD248 antibodies, Clone 8 and 9G5, were generated in house by immunization of rabbits or rats (respectively) with a CD248ECD-Fc fusion protein. These antibodies were selected for all immunostaining and internalization assays as they do not compete with MORAb-004 for CD248 binding in a competition FACS assay (Figure S1C).

### MORAb-004 and CD248 internalization bioassays

To measure MORAb-004 internalization, primary human brain pericytes (5.0xe4) were seeded on 12 well plates for 24 hours prior to beginning the assay. Plates were placed on ice for 5 min and each well was washed once with ice-cold PBS. Each well received 1ml culture medium containing MORAb-004 (5 μg/ml) or monoclonal isotype control antibody (5 μg/ml), followed by incubation for 30 min on ice to prevent CD248 internalization. After cells were washed with ice-cold PBS twice, each well received 1ml of Alexa Fluor-488 conjugated anti-human IgG antibody (Invitrogen) at 2 μg/ml in ice-cold medium and plates were kept on ice for 30 minutes. Unbound antibody was washed off with PBS and then fresh pre-warmed culture media was applied on each well. Then, the plates were transferred to a 37°C incubator. After the indicated incubation time, cells were recovered by either cell dissociation buffer (Invitrogen) to maintain cell surface CD248 or TrypLE buffer (Invitrogen) to remove cell surface CD248. Geometric mean fluorescence intensity value (MFI) of AlexaFluor-488 measured by FACS shows the total intensity of cell surface and internalized CD248 from cell dissociated samples, or a part value of CD248 internalization from TrypLE treated samples, allowing calculation of percent internalization.

To measure CD248 internalization, human brain pericytes were seeded into 96 well black wall clear bottomed poly-D-Lysine coated plates (Greiner bio-one) at 1.5 × 10^4^ cells/well. Serial dilutions of MORAb-004 were prepared in pericyte media from 100 μg/mL to 1.53 ng/mL, and subsequently added to triplicate wells seeded with pericytes, followed by overnight incubation at 37°C, 5% CO_2_. Treated cells were washed with cold 2% BSA/PBS and fixed with cold 0.25% Paraformaldehyde/PBS solution for 20 minutes. The cells were then washed again with cold 2% BSA/PBS and blocked with 1% casein/PBS buffer (Thermo Fisher) for 1 hour. Fixed cells were stained with 0.5 μg/mL biotinylated rat anti-human CD248 IgG 9G5 for one hour. Cells were washed and treated with 1 μg/mL streptavidin-HRP (Jackson IR) for one hour. After a final wash, QuantaBlu fluorogenic peroxidase substrate (Thermo Fisher) was added and fluorescent intensity was measured (325nM excitation, 420nM emission) on a Spectramax M5 plate reader (Molecular Devices).

### Generation of human CD248-knock-in mice

CD248 is a single exon gene and the murine CD248 gene locus was targeted with a full-length human cDNA fragment by homologous recombination (Figure S2A). Homozygous human CD248 knock-in mice were generated on C57BL/6 background through a routine ES cell procedure. The genomic configuration and CD248 expression of the human CD248 was assessed by genomic DNA PCR and RT-PCR with three different primers: one human-murine shared primer HuMs-TEM1-F2-QC (CCCCTACCACTCCTCAGTG), one human allele specific primer Hu-TEM1-R1-QC (CTGGATAGTTGGCTGCGATCAC) and one murine allele specific primer Ms-TEM1-R1-QC (CTGGATAATTGGCCTTGATTTT). Briefly, genomic DNA was extracted from the tails of these mice and control wild type C57BL/6 mice using PureLink^®^ Genomic DNA Mini Kit (Life Technologies) according to the manufacturer's instructions and PCR was performed with the primer sets above. RNA isolation was performed using the PureLink^®^ RNA Purification Kit (Life Technologies). Total RNA was isolated from the lungs of both wild type C57BL/6 and knock-in mice and then RT-PCR was performed with the above primer sets using SuperScript^®^ III One-Step RT-PCR System with Platinum^®^ Taq DNA Polymerase (Life Technologies). All PCR products were analyzed by gel electrophoresis on 3% agarose gels.

### *In vivo* models and MORAb-004 treatment

Homozygous human CD248 (huCD248) knock-in mice or C57BL/6 wide type mice of both genders, 8–10 week old, weights of 18–22g, were used in these studies. For B16-F10 sc model, 5 × 10^5^ cells adapted for *in vivo* growth (B16-F10-T1) were injected into right flank of each mouse. 3 days post implantation, animals (*n* = 15 of each group) were treated daily intravenously for 5 days with MORAb-004 (50mg/kg) or PBS as control. Tumor growth by volume was assessed by three dimensional caliper measurements. For the lung colonization model, B16-F10L1, a variant adapted for lung colonization (B16-F10-L1); or Ll/2-luc-M38-l1, a variant of Lewis lung carcinoma generated *in vivo* from selected lung colonies, were injected intravenously at 1 × 10^5^per mouse, via tail vein. One day prior to cell implantation, animals (*n* = 10 per group) were treated with MORAb-004 or monoclonal isotype control antibody (50mg/kg) intravenously followed by a regimen of every other day treatment post tumor cell implantation. As a control, PBS was administered following the same schedule. For the B16-F10 model, melanoma colony counts in the lungs were recorded as tumor burden. For the Lewis lung carcinoma model, lung colonies were measured via luciferase reporter generated bioluminescence in live animals using IVIS imaging system (Perkin Elmer).

### Microfill perfusion and X-ray micro-CT angiography

B16-F10-tumor-bearing animals received a 50 μl intraperitoneal injection of heparin 10 min before being killed by inhalation of carbon dioxide. The thoracic cavity was opened, an incision was made in the apex of the heart, and a polyethylene cannula (inner diameter, 0.58 mm; outer diameter, 0.96 mm) was passed through the left ventricle and secured in the ascending aorta with a 5–0 silk suture. A 17 ml solution of 0.1 mM sodium nitroprusside in 0.9% saline was perfused at a rate of 6 ml/min^−1^ to provide a state of maximum vasodilatation and to remove blood. MICROFIL (Carver), commercially available lead chromate latex, was prepared as recommended by the manufacturer. Mice were then perfused with 17 ml of MICROFIL at a rate of 2 ml/min^−1^. The infused latex mixture was allowed to polymerize at room temperature for sixty minutes before dissection of tissues of interest. Dissected tumors were immersed in 10% neutral buffered formalin. The tumors were then imaged with a X-ray micro-CT system performed by Numira Biosciences, and analyzed using image analysis software package (Altaview, Numira).

### Immunofluorescent (IF) staining and digital image scanning

5 μm formalin-fixed, paraffin embedded (FFPE) tumor sections were first deparaffinized, then photobleached under white light overnight and finally immersed in EnVision Flex Target Antigen Retrieval buffer (Dako, K8005) in a Lab Vision™ PT Module (Thermo Scientific). Slides were then stained on a Lab Vision™ Autostainer 360 (Thermo Scientific) with designated primary and secondary antibodies. Specimens were mounted and counterstained using mounting medium containing DAPI (VECTASHIELD, H1500). Digital images of each slide were obtained using a Panoramic Midi digital slide scanner (3DHistech, Hungary) and an Olympus IX81confocal microscope.

### Digital quantification of tumor vessel numbers and sizes

Numbers and sizes of tumor vessels in stained tumor sections were quantified using MetaMorph software version 7.7.7.0 (Molecular Devices). Four to six 20X images of representative staining were captured from whole slide scans and digitally analyzed using the Integrated Morphometry Analysis (IMA) function.

### siRNA transfection and TGF-β stimulation

Pericytes (1.5 × 10^6^) were transfected with siRNA specific for CD248 using the P1 Primary Cell 4D-Nucleofector X Kit for the AMAXA (Lonza) electroporation apparatus. siRNA was directed to the following sequence (siRNA2163): CCA ACA AGC GCA TCA CTG A. A sequence specific control siRNA, designed by replacing bases 9 through 11 with their complement, was used as a specific negative control (2163-C911): CCA ACA AGG CGA TCA CTG A. 48 hours after transfection, pericytes were stimulated overnight with 20 ng/mL TGF-β. CD248 and α-SMA protein levels were detected by Western blot analysis and quantification of the Western blot image [Previously described; see [13]].

## SUPPLEMENTARY FIGURES


