# Supplementary material for: Targeting endosialin/CD248 through antibody-mediated internalization results in impaired pericyte maturation and dysfunctional tumor microvasculature
Source: Oncotarget. 2015 Jul 4;6(28):25429–40. doi: 10.18632/oncotarget.4559 (PMC4694842; doi:10.18632/oncotarget.4559)
Supplement: Supplementary file 1 [file oncotarget-06-25429-s001.pdf]

SUPPLEMENTARY FIGURES

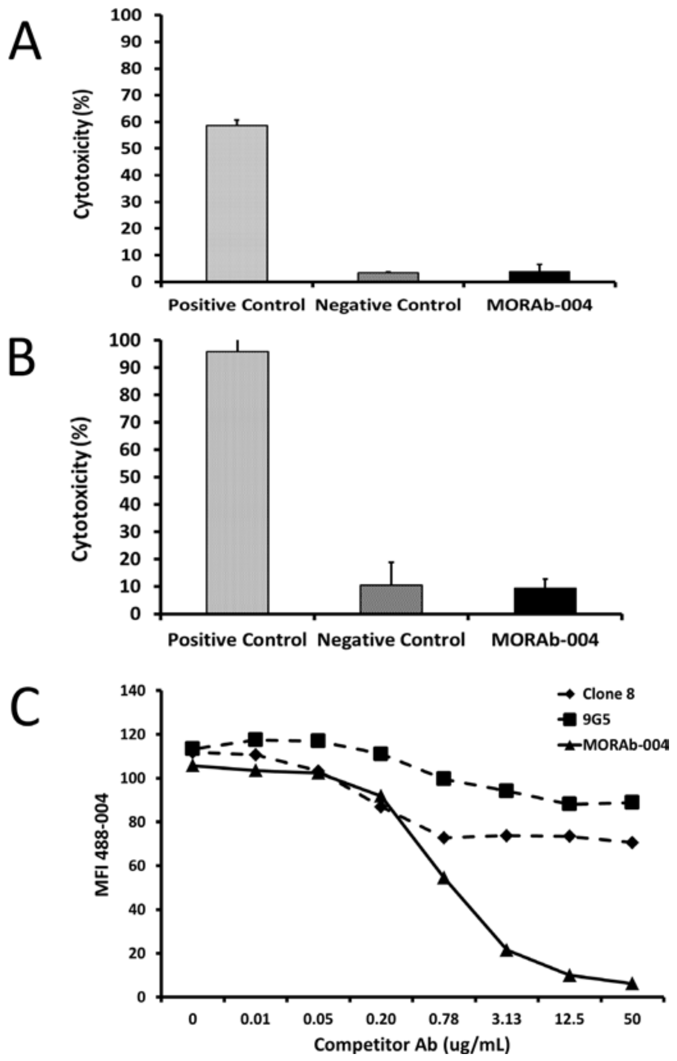

Supplementary Figure S1: Validation of MORAb-004 ADCC and CDC activities and competition binding assay. A, B. Both CDC and ADCC activities of MORAb-004 were evaluated on CHO TEM1 cells. A positive control for these assays was done using CHO K1 cells transfected with folate receptor alpha and a specific antibody with known CDC and ADCC activities. (A) MORAb-004 ADCC on CHO-TEM1 cells. ADCC activity was assessed using an xCELLigence Real Time Cell Analyzer (Roche). CHO-TEM1 cells were incubated in a 96-well E-Plate (Roche) at $2.5x10^4$ cells/well. The plate was incubated at room temperature for 30 minutes per the manufacturer's instructions and then placed into the xCELLigence for 24 hours to acquire readings every hour. Subsequently 100uL of medium was removed and 50uL of either MORAb-004 or a negative control antibody was added to the appropriate wells at 4x the final concentration. The plate was returned to the xCELLigence and incubated for an hour. Human PBMCs (pre-activated with 2ng/mL IL-2 for 24 hours) were added to each well at a PBMC: target cell ratio of 20:1. xCELLigence Cell Index values were measured every 10 minutes for up to 30 hours and percent cytotoxicity at the chosen time point was calculated as follows: [1-(Cell Index with antibody/Cell Index without antibody)]x 100. (B) MORAb-004 CDC on CHO K1/huCD248 cells. For the CDC assay, cells were harvested using Cell Dissociation Buffer (Invitrogen), suspended at $1x10^5$ cells/mL in plain RPMI medium and seeded in a 384-well white plate. Human complement (Quidel) was added to each well with either the testing antibody or the negative control antibody at a final concentration of 10ug/mL and incubated at 37oC for 2 hours. The plate was incubated at room temperature for 30 minutes prior to the addition of Cell Titer Glo to each well following the manufacturer's protocol. The plate was read using a PerkinElmer Fusion microplate analyzer. **C.** Binding competition assay. In house generated anti-CD248 antibodies, Clone 8 (rabbit monoclonal IgG) and 9G5 (rat monoclonal IgG) were screened to ensure that they did not compete with MORAb-004 for CD248 binding in a competition FACS assay. CHO K1 cells transfected with human CD248 (CHO-TEM1 cells) were harvested with cell dissociation buffer (Invitrogen), washed and seeded into a 96-well V-bottom staining plate. Cells were co-stained with a mixture of fixed concentration (0.2ug/ml) Alexa Fluor 488 labeled MORAb-004 and a titration of unlabeled potential competitor antibodies, Clone 8, 9G5 or MORAb-004. Cells were then incubated on ice for 30 minutes. Cells were then washed and fixed with 3% formalin before running on a Millipore Guava Easycyte 8HT. Data is shown as the mean fluorescent intensity (MFI) of Alexa Fluor 488 versus the concentration of potential competitor antibodies.

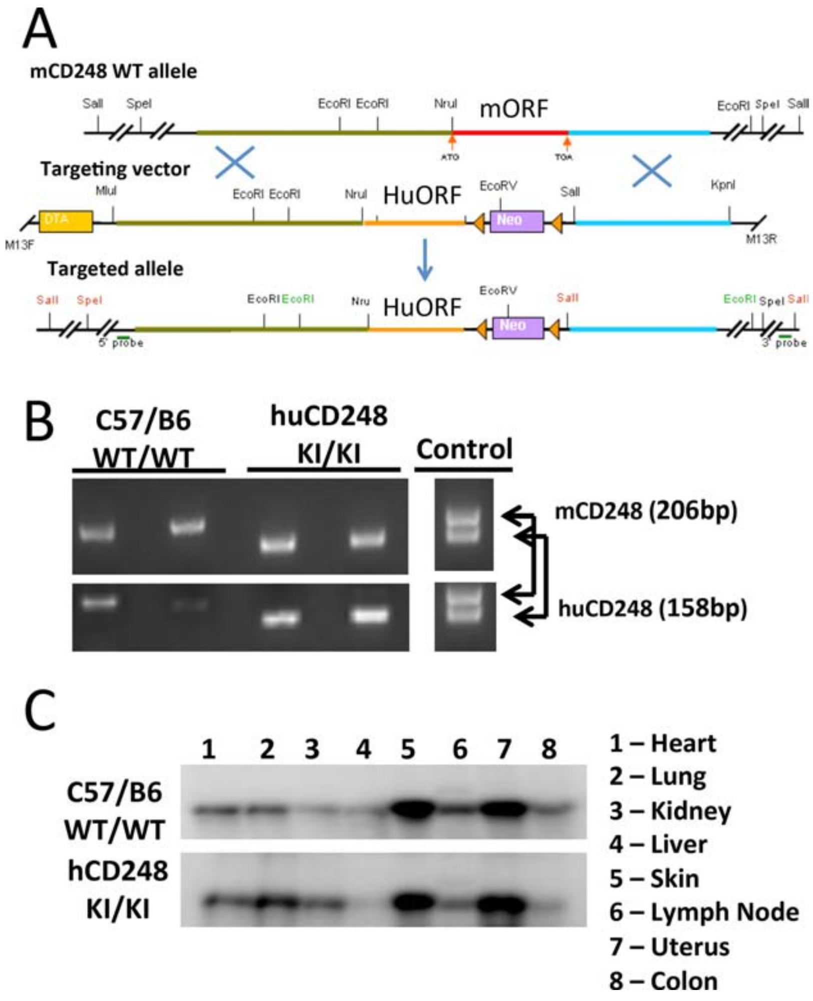

Supplementary Figure S2: Design and validation of human CD248 knock-in mice. A. Design of human CD248 targeting vector and recombination scheme. This design ensured the single ORF of human CD248 gene is completely under control of mouse CD248 promoter. **B.** Genomic and expression validation of the knock-in mice. Genomic PCR (upper panels) and RT-PCR (lower panels). To validate the knock-in mice, three primers were designed based on the alignment of murine and human CD248: one human-murine shared primer HuMs-CD248-F2-QC, one human allele specific primer Hu-TEM1CD248-R1-QC and one murine allele specific primer Ms-CD248-R1-QC. Genomic DNA PCR and RT-PCR with these three primers in the same reaction allowed amplification of either human or murine CD248 gene depending on the template sources. PCR products were distinguished by their sizes where the smaller 158 bp PCR product represented the human allele and the larger 206 bp product represented the murine allele. Genomic DNA PCR and RT-PCR of homozygous human CD248 knock-in and wild type mice indeed gave rise to expected size of PCR products, indicating that the knock-in mice were absent of murine CD248 gene and homozygous for human CD248 gene. PCR control templates are mixed mouse and human CD248 cDNA. **C.** Western blot of tissues from eight major organs from huCD248 knock-in and C57BL/6 wild type mice, were subjected to Western blot analysis to verify proper expression of the human gene in the huCD248 knock-in mice.

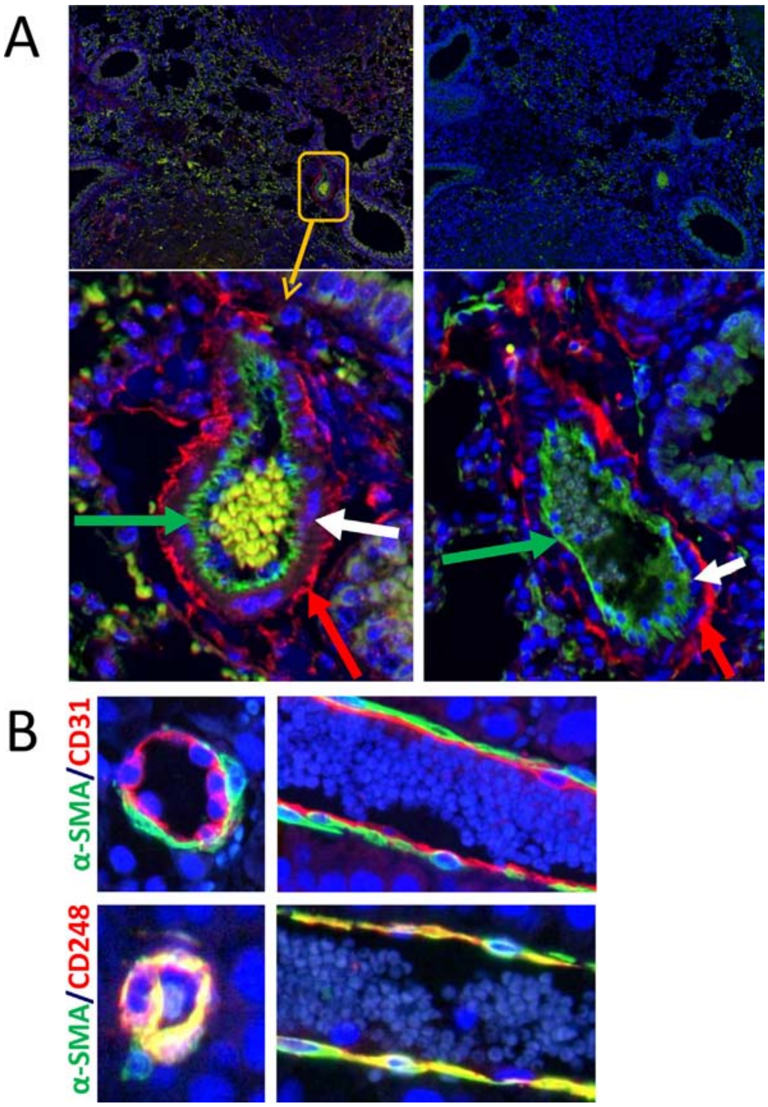

Supplementary Figure S3: Immunofluorescent staining to validate the proper expression of the human CD248 gene in the knock-in mice. A. Immunofluorescent staining of mature blood vessels of normal lung in the wild type mice and huCD248 knock-in mice. Endothelial cells stained with anti-CD31 (green, see green arrows). Both mCD248 and huCD248 were stained with Clone 8, a rabbit anti-TEM antibody that cross-reacts with CD248 from both species (red, see red arrows). Vascular smooth muscle cells were negative for both CD31 and CD248 (see white arrows). Top right panel shows staining performed using monoclonal isotype matched controls for both antibodies on a serial section of tissue to demonstrate the specificity of the staining. **B.** Immunofluorescent staining of neovasculature of B16-F10 s.c. tumor in the huCD248 knock-in mice. α–SMA stained green, while CD31 and CD248 (top and bottom panels, respectively) are stained red.

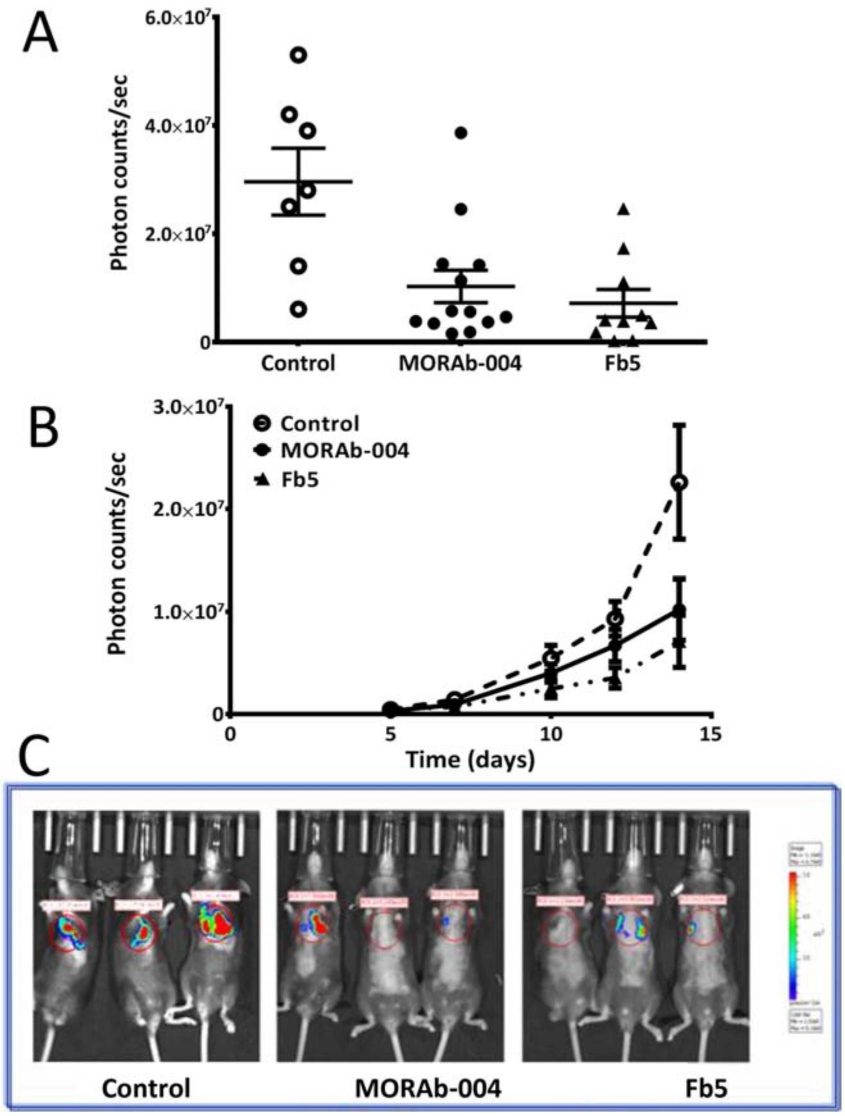

Supplementary Figure S4: MORAb-004 diminished lung colonization of Lewis lung carcinoma in huCD248 knock-in mice. Lewis lung carcinoma cells adapted for *in vivo* growth (Ll/2-luc-M38-l1), at 1×10^5, were injected i.v. vial tail vein into the huCD248 knock-in mice ($n = 10$). MORAb-004, Fb5, or PBS (control) were administered i.v. via tail vein at 50mg/kg 1 day prior to tumor injection and every other day post implantation for a total of 5 doses. Lung colony progression was monitored via engineered reporter bioluminescence using living imaging (IVIS). **A.** Lung colony progression detected via bioluminescence. **B.** Bioluminescence measurements in animals on day 14 prior to lung harvesting. **C.** Bioluminescence images of representative animals from each treatment group.

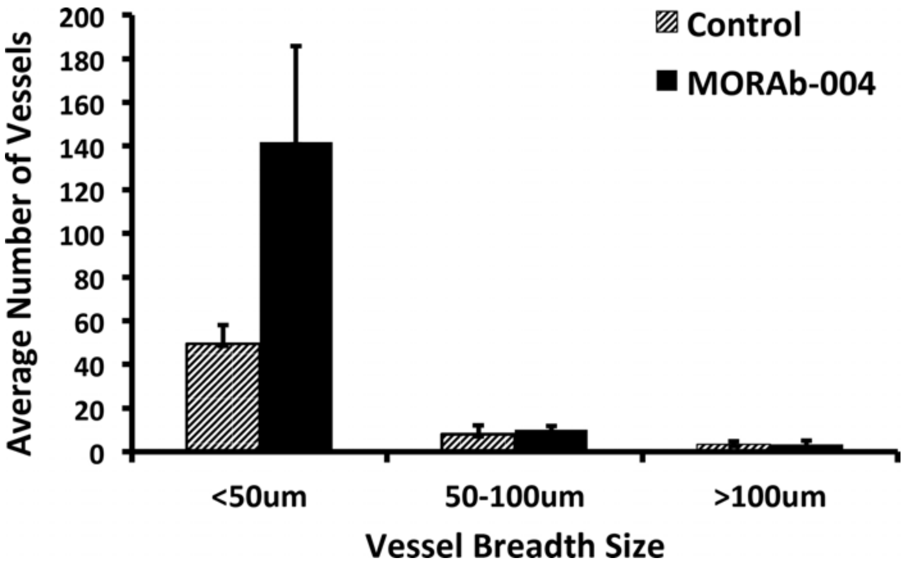

Supplementary Figure S5: Digital analysis of tumor microvessels stained with COLIV and CD31. A. Comparison of digital counting of total number of microvessels in control (PBS) and MORAb-004 treated B16-F10 sc tumor sections stained with ColIV.
